# Original Solution for Middle Ear Implant and Anesthetic/Surgical Management in a Child with Severe Craniofacial Dysmorphism

**DOI:** 10.1155/2015/205972

**Published:** 2015-09-29

**Authors:** Giovanni Bianchin, Lorenzo Tribi, Aronne Reverzani, Patrizia Formigoni, Valeria Polizzi

**Affiliations:** ^1^MD Otolaryngology and Audiology Department, Santa Maria Nuova Hospital, Viale Risorgimento, No. 80, 42100 Reggio Emilia, Italy; ^2^MD Emergency Medicine Department, Santa Maria Nuova Hospital, Viale Risorgimento, No. 80, 42100 Reggio Emilia, Italy

## Abstract

We describe the novel solution adopted in positioning middle ear implant in a child with bilateral congenital aural atresia and craniofacial dysmorphism that have posed a significant challenge for the safe and correct management of deafness. A five-year-old child, affected by a rare congenital disease (Van Maldergem Syndrome), suffered from conductive hearing loss. Conventional skin-drive bone-conduction device, attached with a steel spring headband, has been applied but auditory restoration was not optimal. The decision made was to position Vibrant Soundbridge, a middle ear implant, with an original surgical application due to hypoplasia of the tympanic cavity. Intubation procedure was complicated due to child craniofacial deformities. Postoperative hearing rehabilitation involved a multidisciplinary team, showing improved social skills and language development.

## 1. Introduction

Congenital aural atresia is a general term to describe a spectrum of ear deformities characterized by aplasia or hypoplasia of the external auditory canal. Commonly it is associated with microtia and occasionally with anomalies of the inner ear [1]. This malformation could be associated with other craniofacial dysmorphism and multiorgan dysfunction. It can also belong to different congenital syndromes such as Treacher Collins, Goldenhar, Crouzon, Mobius, Klippel-Feil, Fanconi, DiGeorge, Pierre Robin, Van Maldergem, or other rare diseases, like in our experience [2].

The treatment of congenital aural atresia included first restoration of auditory function and then esthetical reconstruction of the pinna, usually performed not before the age of six [3, 4].

Hearing rehabilitation should begin as soon as possible, to not compromise the development of the language and the social skills. Conventional air-conduction hearing aids are common and easy means to improve patient's deafness. However these devices cannot give an acceptable benefit, if air-bone gaps are as great as up to 60 dB or the external auditory canal is absent or hypoplastic [5]. Besides the application of traditional skin-drive bone-conduction hearing aids, especially in young children, may be complicated by the need to apply constant pressure to the skull to obtain an adequate amplification and it can also represent an aesthetic problem [6]. Modern technology has brought more surgical options and particularly direct-drive bone-conduction devices (BCDs) and middle ear implant have offered an alternative choice for patients suffering from congenital aural atresia [6, 7]. The best solution should be provided after careful multidisciplinary assessment about risks and benefits of all possible treatments [8].

## 2. Case Report

The patient is a 5-year-old child, born at term in the 41st gestational week, from consanguineous parents (first cousins). The pregnancy was complicated by polyhydramnios and fetal ascites. At birth the measurements were as follows: 49 cm length, 2.750 g weight, and occipital-frontal head circumference (OFC) of 35 cm. His past medical history is remarkable for an admission in the Neonatal Intensive Care Unit during the first days of life for respiratory crisis requiring a tracheostomy and a procedure of mandibular advancement by distraction osteogenesis at the age of 2 months for severe micrognathia and retrognathia.

His craniofacial features were distinctive with microcephaly, short palpebral fissures, telecanthus, epicanthus, and bilateral microtia associated with an external auditory canal atresia; limb anomalies included camptodactyly and syndactyly of the fingers and interdigital webbing (diagnosis of Van Maldergem Syndrome) [9, 10].

At the clinical evaluation at the age of five years, speech development was markedly delayed, despite the application of traditional bone-conduction hearing aids from the age of twenty-two months.

The cognitive phenotype showed a psychomotor retardation. Speech production, with the use of hearing aids, was limited to vocalization, babbling reduplicated, and lexical vocabulary of less than ten words. The communication modalities were mostly gestural without clear and evident relational disorders.

To optimize the surgical outcomes, multidisciplinary evaluation of the patient was performed preoperatively.

**Figure 1:**
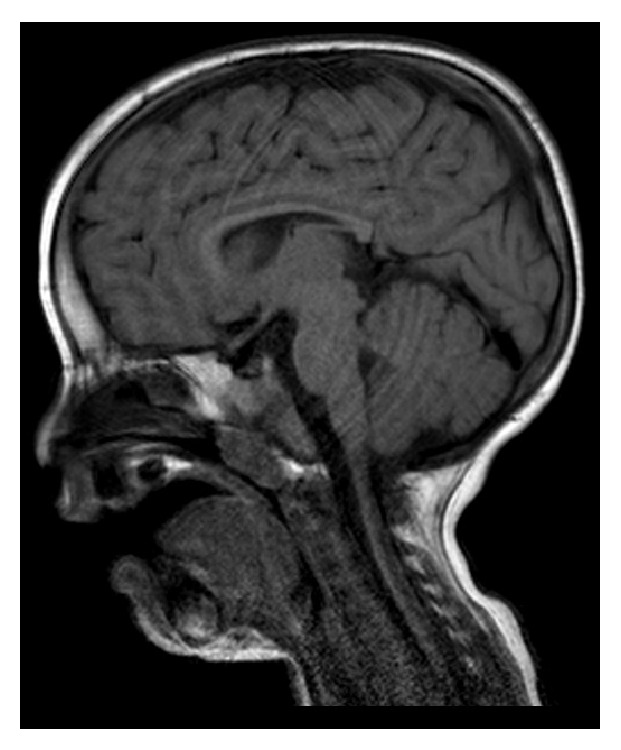
Brain MRI.

Brain MRI revealed neuronal migration abnormalities with hypoplasia of the corpus callosum. The posterior horns of the lateral ventricles were dilated without significant reduction of both hemispheres. Cerebellar abnormalities were not observed (Figure 1).

**Figure 2:**
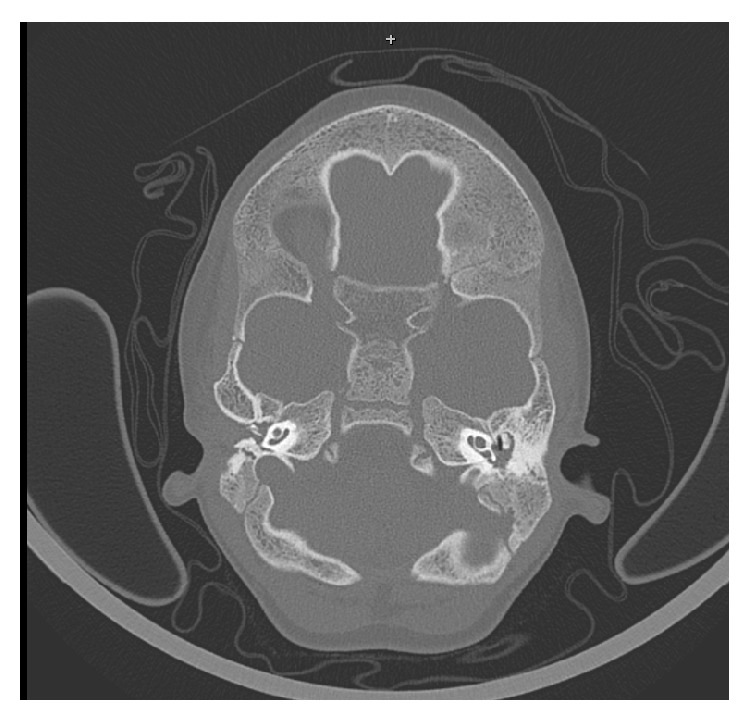
CT scan.

Temporal bone high-resolution computed tomography (CT) showed bilateral external auditory canal atresia, hypoplasia of the tympanic cavity, absent pneumatisation of the mastoid, and radiological normal cochlear morphology. No clear discontinuity and malformation of the ossicular chain have been revealed (score 6 according to the Jahrsdoerfer classification of congenital aural atresia) (Figure 2).

A preoperative pure-tone audiometric test showed a bilateral conductive hearing loss (AC-PTA: 70 dB HL; BC-PTA: 20 dB HL). We have detected a good functional gain with hearing aids although the patient did not have a good compliance and the device was not used correctly. This has influenced not only communication skill but also some aspect of quality of life such as peer-acceptance and self-esteem because of his physical appearance, like this was reported by his family.

In agreement with the parents, the boy was submitted to Vibrant Soundbridge (VSB) implantation [7–11]. The choice was made considering age, medical history, anesthetic risk, radiological aspect, and parents' expectations.

Vibrant Soundbridge (VSB) is a middle ear implant composed of two parts, the external audio processor (AP) and the implantable Vibrating Ossicular Prosthesis (VORP), consisting of the internal receiving coil, a modulator, a cable, and the floating mass transducer (FMT), applied to one of the vibratory structures of the middle ear [12, 13].

The child underwent surgery under general anesthesia, with the use of a facial nerve monitoring system. Maxillofacial dysmorphism posed technical difficulties to the anesthesiologists for the safe management of the airway. Intubation was carried out by two anesthetists, side by side; while the first introduced the laryngoscope into the oral cavity (lidocaine 4% spray was added to minimize the instrument response), without loading the tongue, the second one introduced the pediatric fiberscope (2.8 mm diameter) on which the armed cuffed tracheal tube was loaded and displayed and exceeded the glottis. The procedure was very rapidly completed at the first attempt, maintaining the patient in spontaneous breathing, and the armed orotracheal tube was correctly positioned. During surgery any other anesthetic complication was not observed [14].

The Vibrant Soundbridge was implanted on the left side, using a posterior approach [15]. A hairline incision was making through all layers behind a safety area surrounding ear position. Then a muscle-periostal flap was raised towards the atresia plane and a “pocket” was created to insert the internal receiving coil. A mastoidectomy and a posterior tympanotomy were performed to ensure adequate exposure of the middle ear space. Due to hypoplasia of the tympanic cavity, there was not enough space for the correct placement of the FMT on the round window (RW vibroplasty technique) or in contact with the incudostapedial joint onto the incus (incus vibroplasty) [16]. Suddenly the facial nerve was dehiscence so the crimping of the clip on the stapedial crus was not available.

**Figure 3:**
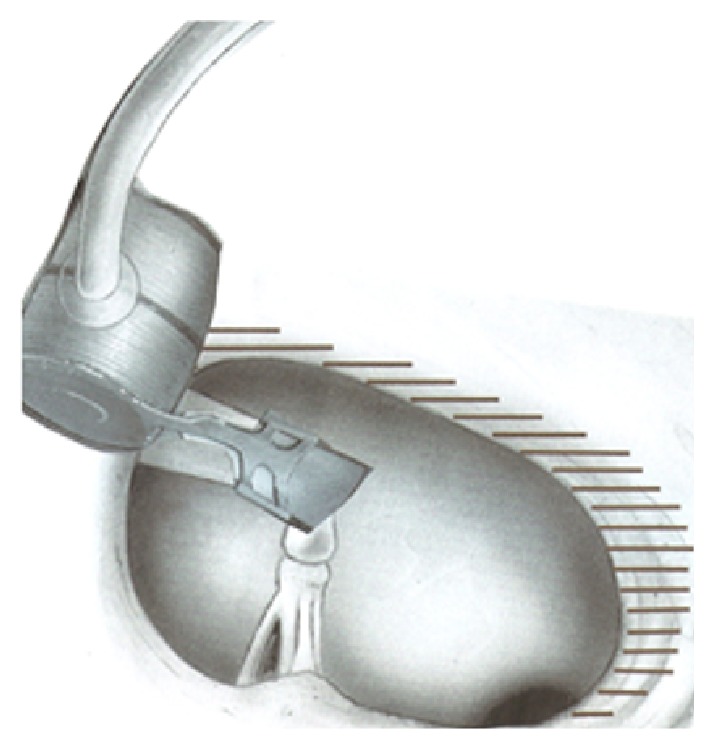
Middle ear implant application. The FMT was placed to the long apophysis of the incus with superior orientations.

Finally the FMT was placed to the long process of incus with superior orientations (Figure 3).

Care has been taken that the axis of the FMT was put parallel to the orientation of the stapes, so that the device vibrated simulating the natural movement of the ossicular chain. This type of technical solution is also compatible with the new model of Vibrant Soundbridge (VORP 503 with vibroplasty couplers). Intraoperative test was performed and the device was working properly.

The VSB was activated eight weeks after surgery and no postoperative complications were observed as well as facial nerve injury or inner ear damage. A good improvement in perception scores was noted after one year using speech perception test conducted with no visual contribution in free field (Table 1).

An open questionnaire regarding the use of the VSB and the change in social life before and after surgery after one year was compiled by the parents. The device was well accepted and used properly by the young patient probably because VSB is less visible and more comfortable.

## 3. Discussion

Bone-conduction devices (BCDs) and middle ear implants offer new treatment options in children with congenital aural atresia when the conventional hearing aids are not successful [8].

A careful multidisciplinary assessment, which includes audiological and radiological evaluation, clinical history, and anesthetic risk, helps to identify the best surgical solution.

In this case, the child suffered from a rare disease, Van Maldergem Syndrome, and craniofacial dysmorphism posed a significant challenge for both the anesthesiologist and the surgeon.

All hearing devices, available at that time, have been evaluated.

A bone-anchored hearing aid (BAHA implantation) was proposed as first option. This is a percutaneous bone-anchored device that consists of an external sound processor that converts sound energy into vibration through a percutaneous abutment to the skull. The main indications are a minimum of five years of age at the time of implantation and a cortical bone thickness >3 mm. This technique is a reversible surgical procedure that avoids any risk of additional hearing loss in the patients. However parents were informed of the possible postoperative risks of skin complication, fixture loss, and osseointegration failure, which have a higher incidence in children, in particular in syndromic one as reported in literature [17]. Bonebridge implant was evaluated as second option. This device consists of an external part, the audio processor, and an implanted part, the bone-conduction implant (BCI). It uses a bone-conduction floating mass transducer (BC-FMT) which is surgically fixed with two screws in the temporal bone and completely covered by the skin. Sound received by the external processor is transmitted to the BCI transcutaneously via an electromagnetic field. The BC-FMT has a diameter of 15,8 mm and height of 8,7 mm and an area of the skull with thickness of at least 3,9 mm is needed, to fix the two screws into the temporal bone. This device was approved only for patients >18 years, even if there were preliminary studies in younger children reported in the literature [18]. In this case TC imaging and cranial anomalies excluded this surgical option due to the malformation of the skull and the thickness of the bone.

Lately the Vibrant Soundbridge extended its indications to patients younger than eighteen years, providing another surgical option for bilateral congenital conductive or mixed hearing loss [11].

This hearing device, unlike BAHA and Bonebridge, provides stimulation to the inner ear in a different way through an electromagnetomechanic cylinder (FMT) placed to the ossicular chain or to the oval or round window. The external processor picks sound signals up and transmits them to the internal receiver/demodulator as electrical signal. The demodulator transmits the information to the FMT that provides direct stimulation of the cochlea.

The position of the floating mass transducer depends on the middle ear anatomy. A high-resolution computer tomography (HRCT) should be routinely used preoperatively to assess the bony structures of the temporal bone and tympanic cavity [19]. In this case abnormal tympanic facial nerve course and the hypoplasia of middle ear space did not allow the placement of the FMT onto the long process of the incus in the classical way. However the novel placement, adopted according the anatomical situation, showed good performance, as intraoperative test has proven. This technical solution allowed the correct vibration of the FMT, ensuring a viable alternative in cases of a limited space of the tympanic cavity. Treatment choice was made evaluating risks and benefits of each treatment option and considering parents opinion.

## 4. Conclusion

Modern technology has brought more surgical options. Bone-conduction devices (BCDs) and middle ear implant offer an alternative choice for patients suffering from congenital aural atresia. In particular in children with additional disabilities a careful multidisciplinary assessment is important, considering risks of all possible treatments to provide benefits in social and relational skill that are important for their quality of life.

## Figures and Tables

**Table 1:** Audiological evaluation before and after surgery.

Speech perception test	Preoperative	Postoperative (with VSB) after one year	
(1) Vocal recognition	30%	100%	
(2) Word recognition	0%	70%	
(3) Sentence recognition	0%	76%	

Pure-tone audiometric test in free field	Without hearing aid	With traditional bone-conduction hearing aid	With VSB

	70 dB HL	35 dB HL	25 dB HL
